# The Company of Biologists: a century in review

**DOI:** 10.1242/bio.062413

**Published:** 2025-12-19

**Authors:** O. Claire Moulton, Saanjbati Adhikari, Katie Ward

**Affiliations:** ^*^ Chief Executive Officer, The Company of Biologists; ^‡^ Reviews Editor, Development; ^§^ Chief Financial Officer and Charity Director, The Company of Biologists

The year 2025 marks a significant milestone in the history of The Company of Biologists and we have enjoyed an extraordinary year of celebration. We now take the opportunity to look back on activities that included our Biologists @ 100 conference in March and the content we've published throughout the year to celebrate both our publishing and charitable activities. At the same time, we share a new strategic plan for The Company of Biologists with a focus on supporting biologists and inspiring biology – for the next 100 years.

## A unique conference to celebrate community

A highlight of our anniversary year was the Biologists @ 100 conference, which took place on 24–27 March in Liverpool. The meeting brought together researchers from across the disciplines represented by our five journals: Development, Journal of Cell Science, Journal of Experimental Biology, Disease Models & Mechanisms, and Biology Open (BiO). It was held in partnership with the three societies we help support through our charitable funding – the British Society for Cell Biology (BSCB), British Society for Developmental Biology (BSDB) and the Society for Experimental Biology (SEB). Altogether, we welcomed 585 attendees from 27 countries (and six continents), and featured research from 76 speakers and 286 posters.

The conference programme offered a unique opportunity to attend a diverse range of sessions. Topics were chosen to pique people's curiosity to learn something new in a talk they might not normally have chosen. And in our plenary sessions – on climate change and biodiversity loss, health and disease, and emerging technologies – we wanted to tie together this curiosity-driven approach by setting our conference themes within a broader context, focusing on key challenges and opportunities facing biologists, and the world, today (https://www.biologists.com/100-years/conference/videos). We thank our scientific organisers, Sarah Bray, Steve Clapcote, Craig Franklin, Steve Royle, Holly Shiels and Jim Smith, for their contributions to this interdisciplinary scientific programme.

The event itself was a showcase for sustainable conferencing, with carbon-conscious choices embedded in everything from venue selection and delegate travel through to plant-based catering and a sustainability zone. We hosted author focus groups at the conference – asking the community to help us shape our journals for the future by sharing their publishing priorities, highlights and concerns. We took the opportunity to preview our Event Carbon Calculator (https://www.biologists.com/sustainability-hub/event-carbon-calculator/), which helps meeting organisers reduce the carbon footprint of their events. We were also delighted to be joined by the Woodland Trust, who support our biodiversity initiative, The Forest of Biologists (Moulton and Freeman, 2023), which was originally conceived by former Editor-in-Chief (EiC) of BiO, Steven Kelly (University of Oxford, UK), who said, ‘The whole Company got on board eventually, and we ended up rolling it out company wide. It has been incredibly rewarding to watch the forest grow and to see the impact it has had on people, the landscape, and biodiversity’.

## BiO articles over the course of 2025

**Fig. 1.**
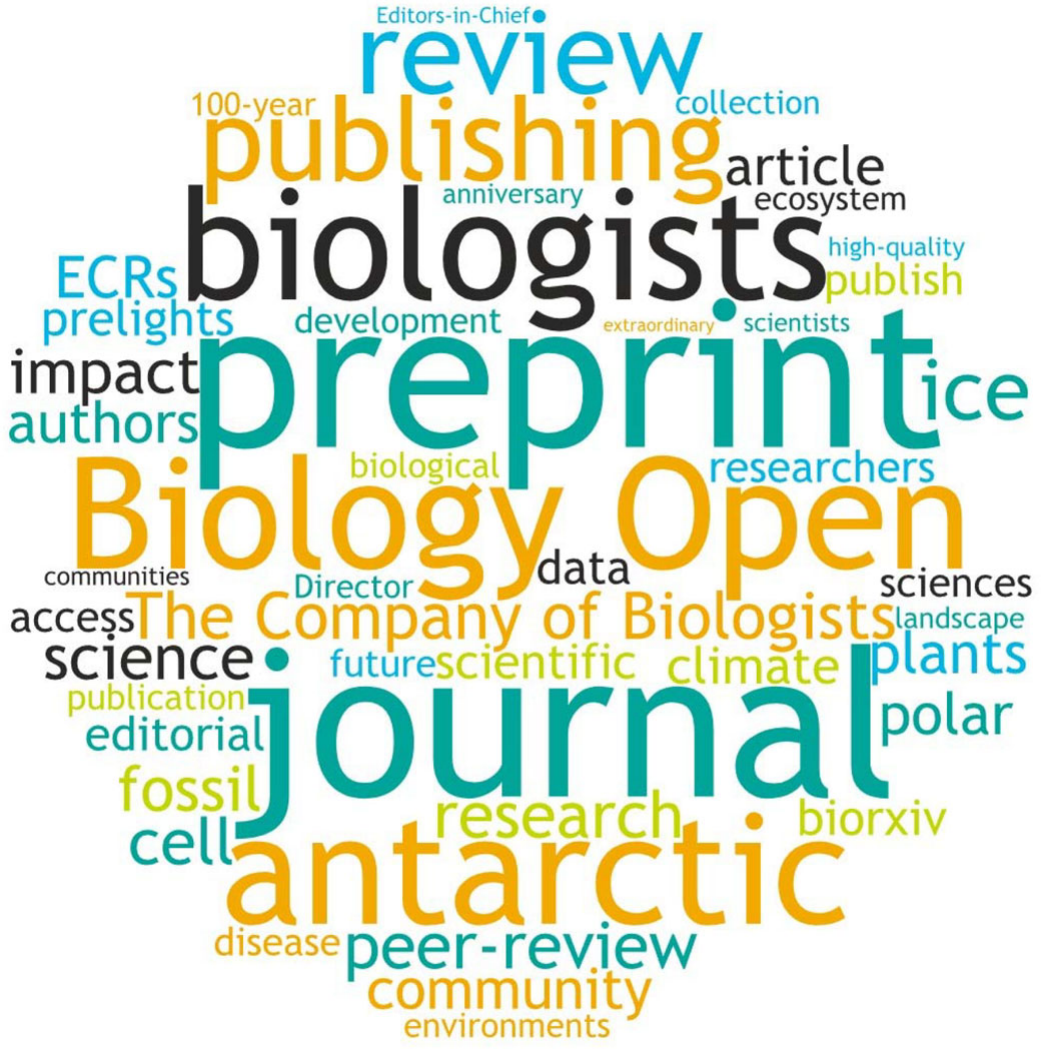
**A word cloud showing a selection of the most frequently used words from articles published in BiO's anniversary article collection.** Generated with https://www.wordclouds.com/.

Over the past year, BiO has published articles across a few key categories – publishing, charity and community – as part of our 100-year anniversary subject collection (Fig. 1). The year began with some of our Directors reviewing the journey of The Company of Biologists (Bray et al., 2025) and, in the months that followed, the past and present EiCs of BiO shared their experiences of being involved with the journal and their aspirations for BiO's future (Adhikari et al., 2025). We also learned about what fossil plants can tell us about climate, the importance of Antarctic research in understanding our changing world, and the benefits of open science from Jane Francis, Director of the British Antarctic Survey, who was a plenary speaker at the Biologists @ 100 conference (Pickup, 2025).

We have also stayed ahead of the publication curve by embracing preprints long before it became a trend followed by other publishers. To this end, we published an article that reflects on the ‘transformation of the preprint landscape since 2013 and how the Company has adapted to and embraced this (new) way of sharing research’ (Prosée and Brown, 2025). We also caught up with a few BiO authors from across the journal's history to learn about their initial impressions of BiO and its impact on their careers (Adhikari, 2025).

Making 2025 a year full of activity and celebration has relied on enthusiasm and effort from every member of our staff here at The Company of Biologists, including all our departments from Charity to Production, from Sales and Marketing to Accounts, and from Events to Editorial. We sincerely thank everyone for their contributions and you can learn more about some of our staff in our recent story https://www.biologists.com/stories/the-people-behind-the-company-of-biologists/. We also thank our colleagues who have collaborated across our five journals and three community sites to produce our anniversary content: Saanjbati Adhikari, Katherine Brown, Alejandra Clark, Amelia Glazier, Seema Grewal, Rachel Hackett, Michaela Handel, Kirsty Hooper, Kathryn Knight, Dina Mikimoto, Andrea Murillo and Helen Zenner. In addition, we thank the 100-anniversary project team, Jitske de Vries, Jane Elsom, Alex Eve, Claire Moulton and Katie Ward, who have overseen this anniversary year and all the ventures therein.

## Our extraordinary community

Throughout the year, we spotlighted 100 biologists with an extraordinary link to the Company who have made an important contribution to biology (https://www.biologists.com/100-years/100-biologists/; Box 1). ‘Extraordinary’ has many faces and it was difficult to restrict ourselves to 100 people, but this was a wonderful way to recognise a wide range of biologists from different career stages, locations and periods in history. Look out for our founder, the marine biologist George Parker Bidder III, in the collection, who at one point held the world record for the oldest message in a bottle (stemming from his work on ocean currents). To honour him, we set up our own ‘message in a bottle’ project. We've been gathering stories from you, our community, on how The Company of Biologists has supported you over time and how that has positively impacted your careers. We're highlighting a selection of stories, and we're delighted to hear from those who've published a key paper in one of our journals or contributed to one of our community sites, and of course those who've worked with us as Directors, Editors, or on one of our other projects.

Box 1. The ‘100 extraordinary biologists’ we have featured during 2025 Michael Abercrombie

Saanjbati Adhikari

Aymen al-Rawi

Rohini Balakrishnan

Daniel Ríos Barrera

Renata Basto

Kênia Cardoso Bícego

George Parker Bidder III

Anahí Binagui-Casas

Bob Boutilier

Andrea Brand

Sarah Bray

James Briscoe

Katherine Brown

Carol Bucking

Clotilde Cadart

Paul Conduit

Kim Cooper

Mariana De Niz

Gautam Dey

Michael Dickinson

Jeroen Dobbelaere

Katherine Duncan

Sadaf Farooqi

Tony Farrell

Honor Fell

Jane Francis

Craig Franklin

Andrea Fuller

Joachim Goedhart

Daniel Gorelick*

John Gurdon

Amanda Haage

Cathy Jackson

Helena Jambor

Monica Justice

Steve Kelly*

August Krogh

Tamina Lebek

Christophe Leterrier

Ottoline Leyser

James Liao

Grace Lim

Jennifer Lippincott-Schwartz

Sally Lowell

Emily Lucas

Simon Maddrell

Tshepiso Majelantle

Elaine Mardis

Paul Martin

Peter Medawar

Pleasantine Mill

Mole

Claire Moulton

Andrea Murillo

Janni Nüesslein-Volhard

Godwin Pius Ohemu

Guangshuo Ou

Hoover Pantoja-Sanchez

Sheila Patek

Andrea Paterlini*

Liz Patton

Mark Peifer

Norbert Perrimon

Daisy Pineda-Suazo*

Hans-Otto Pörtner

Manu Prakash

Reinier Prosée

Jordan Raff*

Liz Robertson

Nadia Rosenthal

Janet Rossant

Owen Sansom

Martin Schwartz

Holly Shiels

Vivian Siegel

Zolelwa Sifumba

Austin Smith

Jim Smith

Arnoud Sonnenberg

John Speakman

David Stephens

Kate Storey

Charles Swanton

Stephen Tait

John Treherne

Sylvie Urbé

Teresa Valencak

K. Vijayraghavan

Tobias Wang

Jennifer Waters

Fiona Watt

Michael Way

Vincent Wigglesworth

Caroline Williams

Chris Wood

Chris Wylie

Martha S. C. Xelhuantzi*

Helen Zenner

Meng Zhu

Names with asterisks indicate biologists with specific links to BiO.

At the beginning of the year, Daniel Gorelick (Baylor College of Medicine, USA), the current EiC of BiO, reflected on what motivated him to take on the role. ‘I am passionate about improving scientific publishing, particularly the peer review process,’ he explained, ‘I saw the position as an opportunity to put ideas into practice that could make peer review faster, fairer and more transparent’. Gorelick emphasised that he envisions BiO as a journal recognised for ‘cost-effective, high-quality peer review with rapid turnaround – publishing manuscripts where the conclusions are supported by the data, irrespective of impact’ (Adhikari et al., 2025).

Authors publishing in BiO agree that their experience has been both time- and cost-effective. ‘When BiO was launched, I was enthusiastic about supporting its mission to evaluate scientific work based on its impact within the community, rather than relying solely on individual or combined metrics’, said Michael Hebert (University of Mississippi Medical Center, USA), who has published ten articles in the journal to date.

‘[BiO] launched several initiatives to support ECRs, from internships to our Future Leader and A Year at the Forefront writing programmes, as well as funding ECR-led conferences and events’, shared Steven Kelly. Indeed, Andrea Paterlini (University of Edinburgh, UK), who published a Review in BiO in 2020 (Paterlini, 2020) and interviewed as a part of our Future Leaders to Watch series, recalled, ‘I was just finishing my PhD at the time, and I jumped at the opportunity because showcasing your thinking and gaining some visibility can be very valuable for ECRs’. Paterlini shared that publishing with BiO was ‘so smooth’ that he ‘decided to go through the entire process a second time’, publishing another Review (Paterlini, 2023) as a group leader (Adhikari, 2025).

Similarly, Daisy Pineda-Suazo (Universidad Nacional Autónoma de México, Mexico), who was recently interviewed for our First Person series after her first-author publication in BiO (Pineda-Suazo et al., 2024), shared, ‘My colleagues, who had previously published in the journal, recommended BiO for its rigorous but supportive peer-review process. Additionally, its Open Access (OA) format and focus on high-quality biological research made it an attractive option for disseminating my findings to a broader scientific audience’.

It was the BiO transfer network that enabled Albena Dinkova-Kostova (University of Dundee, UK) to publish in BiO. ‘When we submitted the paper to Journal of Cell Science, the Editors recommended transferring it to BiO – a then-new journal that focused on scientific rigour and sound methodology, rather than emphasising wide appeal’, said Dinkova-Kostova, whose corresponding author paper is now the most-cited BiO article (Holmström et al., 2013).

‘Our main aim was to publish our manuscript in an OA journal that reaches a wide community across the biomedical sciences’, echoed BiO author Wim van der Poel (Kontos et al., 2022; Adhikari, 2025).

As the Company steps into the next century, BiO took a bold step earlier in 2025 to change the traditional peer-review process with our Fast & Fair peer review initiative, ‘an experiment designed to deliver rigorous, high-quality peer review within seven business days’ (Gorelick and Clark, 2025). BiO's ethos will remain to ‘benefit the research community by rapidly disseminating rigorous and reliable science in a transparent manner’ in the coming decades (Adhikari et al., 2025).

## Looking ahead – internal changes

**Fig. 2.**
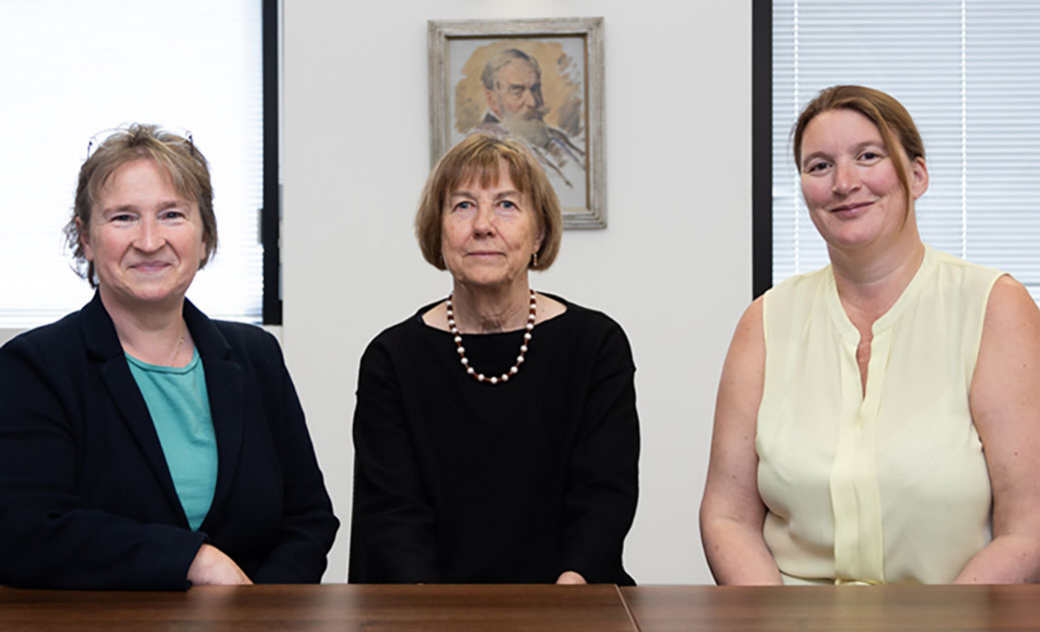
**A new structure for a new century.** From left to right: Claire Moulton (Chief Executive Officer), Sarah Bray (Chair of the Board of Directors) and Katie Ward (Chief Financial Officer and Charity Director).

The anniversary year also saw internal transformation as the Company turned its thinking towards the future. Claire Moulton was appointed as the Company's first Chief Executive Officer, with Katie Ward being appointed as Chief Financial Officer, reflecting a commitment from the Board of Directors to harness our in-house publishing expertise and promote collaborative thinking across the organisation (Fig. 2).

Our new strategic plan (2025-2028) focuses on four key areas: (1) Work together across all areas of the organisation to support the biological community, including building on our relationships with societies and other philanthropic organisations. (2) Strengthen our journals as highly respected publications that support the advancement of biological research while preserving the unique qualities of our journals, providing a great author experience and building trust and transparency in our publishing practices. (3) Develop a sustainable pathway to support the Company's activities for another 100 years, covering both financial sustainability and environmental sustainability. (4) Build on our reputation for community-focused innovation – 2025 featured BiO's Fast & Fair peer review initiative and the sustainability team's event carbon calculator.

As the anniversary year draws to a close, The Company of Biologists has had a lot to celebrate. The next 100 years give us even more to think about as we continue our commitment to supporting biologists and inspiring biology.
